# Expression of an Organic Solvent Stable Lipase from *Staphylococcus epidermidis* AT2

**DOI:** 10.3390/ijms11093195

**Published:** 2010-09-13

**Authors:** Raja Noor Zaliha Raja Abd. Rahman, Nor Hafizah Ahmad Kamarudin, Jalimah Yunus, Abu Bakar Salleh, Mahiran Basri

**Affiliations:** 1 Faculty of Biotechnology and Biomolecular Sciences, Universiti Putra Malaysia, Serdang, Selangor, Malaysia; E-Mail: hafizah.kamarudin@gmail.com (N.H.A.K.); 2 Institute of Biosciences, Universiti Putra Malaysia, Serdang, Selangor, Malaysia; 3 Faculty of Sciences, Universiti Putra Malaysia, Serdang, Selangor, Malaysia

**Keywords:** Staphylococcus epidermidis, prokaryotic system, organic solvent stable

## Abstract

An organic solvent tolerant lipase gene from *Staphylococcus epidermidis* AT2 was successfully cloned and expressed with pTrcHis2 in *E. coli* TOP10. Sequence analysis revealed an open reading frame (ORF) of 1,933 bp in length which coded for a polypeptide of 643 amino acid residues. The polypeptide comprised of a signal peptide (37 amino acids), pro-peptide and a mature protein of 390 amino acids. Expression of AT2 lipase resulted in an 18-fold increase in activity, upon the induction of 0.6 mM IPTG after a 10 h incubation period. Interestingly, this lipase was stable in various organic solvents (25% (v/v), mainly toluene, octanol, p-xylene and *n*-hexane). Literature shows that most of the organic solvent stable bacterial lipases were produced by *Pseudomonas* sp. and *Bacillus* sp., but very few from *Staphylococcus* sp. This lipase demonstrates great potential to be employed in various industrial applications.

## 1. Introduction

Lipases are a class of enzymes that catalyze the hydrolysis of long chain triacylglycerols. The importance of microbial lipases like these from Staphylococcal proteins, is not only in their involvement in some pathogenic processes but also in industrial sectors due to their ability to catalyze many reactions based on chain length selectivity, regiospecificity, and chiral selectivity [1]. These days, the ability of an enzyme to retain its activity in the presence of organic solvents is an attractive property, as many reaction media for enzymatic reactions involve the use of organic solvents. Organic solvents are basically known to be toxic to most bacteria as it compromises the structural and functional integrity of the cell [2,3]. Organic solvent tolerant bacteria exhibit certain adaptations to circumvent the toxic effect, such as, by having solvent efflux pump, rapid membrane repair, decreased cell surface hydrophobicity, lower cell membrane permeability and increased membrane rigidity. Inoue and Horikoshi (1989) made the first discovery of organic solvent stable bacterium, a strain of *Pseudomonas putida* IH-2000 which could actively grow and multiply in the presence of 50% (v/v) toluene [4]. The enzymes produced by these organic solvent tolerant microbes are logically stable in a solvent-rich environment [5]. The earliest study on such an organic solvent tolerant lipase was reported by Ogino *et al.*, (1994) wherein the lipolytic activity of *P. aeroginosa* LST-03 increased in the presence of toluene, cyclohexane, ethanol and acetone [6]. Since then, many organic solvent tolerant lipases were isolated; mainly from *Pseudomonas* and *Bacillus*. Previously, seven putative lipase producers were isolated from contaminated soil at a car service station in Port Dickson, Negeri Sembilan, Malaysia. Among them, isolate AT2, with the highest lipase production of 0.2937 U/mL, was selected for further study. This isolate was found to be stable in 40% (v/v) BTEX (benzene, toluene, ethyl-benzene and xylene) and identified as *S. epidermidis* AT2 [7]. In this paper, the cloning and expression of this organic solvent-tolerant lipase by PCR technique and its stability in organic solvents was studied. To date, only one report on organic solvent tolerant lipase isolated from *S. epidermidis* is available [8].

## 2. Results and Discussion

### 2.1. Cloning and Nucleotide Sequence of the Lipase AT2 Gene

**Figure 1:**
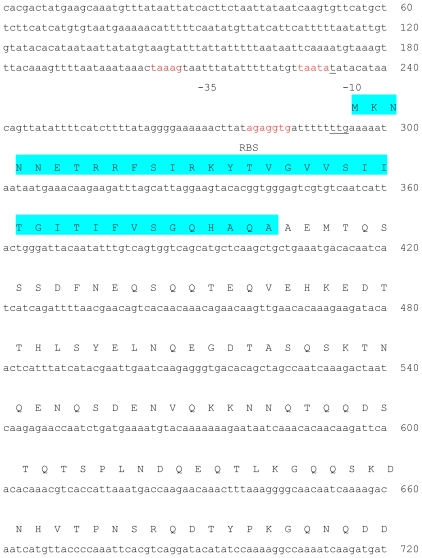

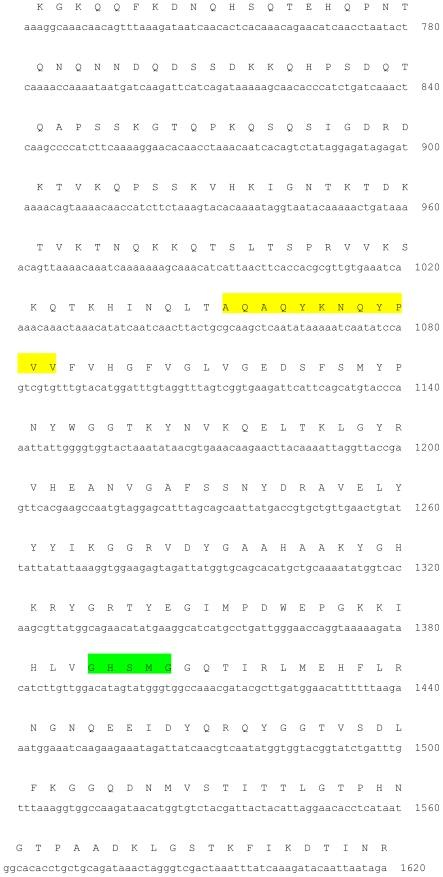

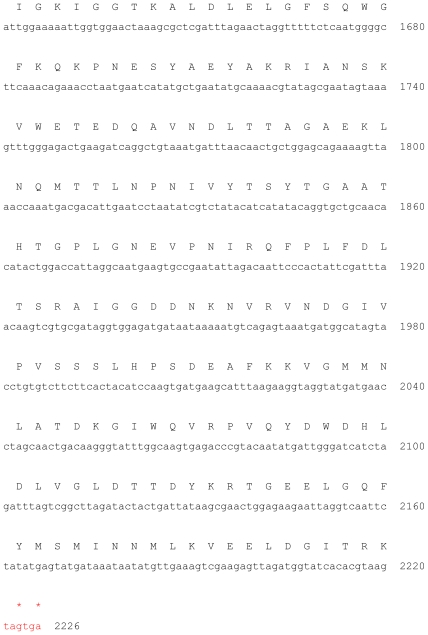
Nucleotide and deduced amino acid sequences of the organic solvent tolerant lipase gene of *S. epidermidis* AT2. Note: The predicted promoter region (−10 and −35 promoter) and ribosome binding site (RBS) are highlighted in red and part of the mature enzymes are highlighted in yellow. The signal peptide is highlighted in blue. The pro-peptides are located in between the signal peptide and mature part. The conserved pentapeptide is highlighted in green, start and stop codon are underlined and indicated by (*), respectively. The AT2 lipase sequence has been submitted to GenBank database under the accession number EU814893.

The entire 2.3 kb containing the putative lipase gene was sequenced and found to contain a single open reading frame (ORF) comprising 1933 bp extending from 293 to 2226 (Figure 1), which coded for 643 amino acids. From the analysis of the nucleotide sequence data, the translation initiation codon was predicted to be TTG. The same finding was also reported for other Staphylococcal proteins including *gehD* [9]. The initiation codon was found to be preceded by a potential ribosome-binding site, AGAGGTG, identical to that found upstream of *gehD*. According to sequence comparisons, staphylococcal lipases are generally translated in the form of pre-pro-enzyme; comprising of signal peptide, pro-peptide and mature active form [10]. The *N*-terminal pre-peptide or signal peptide of *S. epidermidis* AT2 lipase consisted of 37 amino acids (Met to Ala). The amino acids sequence which coded for the signal peptide were; –′M K N N N E T R R F S I R K Y T V G V V S I I T G I T I F V S G Q H A Q A′–. The function of signal peptides is mainly in the transport of proteins and the secretion pathway. The putative signal peptide cleavage site was located in between Ala-37 and Ala-38 when predicted by using the SignalP V2.0 World Wide Web server. The pro-peptide sequence consisted of 216 amino acids; located in between the signal peptide and mature AT2 lipase form (Ala-38 to Thr-216). The role of pro-peptide was still unclear, however, according to a study on *S. hyicus* lipase, the pro-peptide involved in stabilization against proteolysis and protein translocation [11,12] The deduced molecular mass and pI, based on the ORF were calculated to be 72.2 kDa and 8.1, respectively. The complete sequence of the ORF of AT2 lipase gene was submitted to GenBank and assigned an accession number EU814893. The nucleotide sequence of the open reading frame of the recombinant AT2 lipase had a very high homology with that of the lipase *gehD* of *S. epidermidis* 9 (99%). Therefore, *S. epidermidis* AT2 was identified as a lipase producer. The nucleotide sequence of the structural gene of AT2 mature lipase and its nearby sequences were the same as those of lipase of *S. epidermidis* 9 (AF090142)*, S. epidermidis* RP62A*, S. xylosus* (AF208229), *S. hyicus* (X02844), *S. aureus* (M12715) and *S. warneri* (BAD90265) except for some nucleotides. However, none of these lipases were reported to be organic solvent tolerant. Multiple alignments of the amino acid sequences of lipase AT2 with those lipases from *S. aureus, S. haemolyticus* and *S. hyicus* showed that the translation products consisted of three distinct domains which corresponded well with the predicted locations of the signal peptide, pro-peptide and mature lipase.

**Figure 2:**
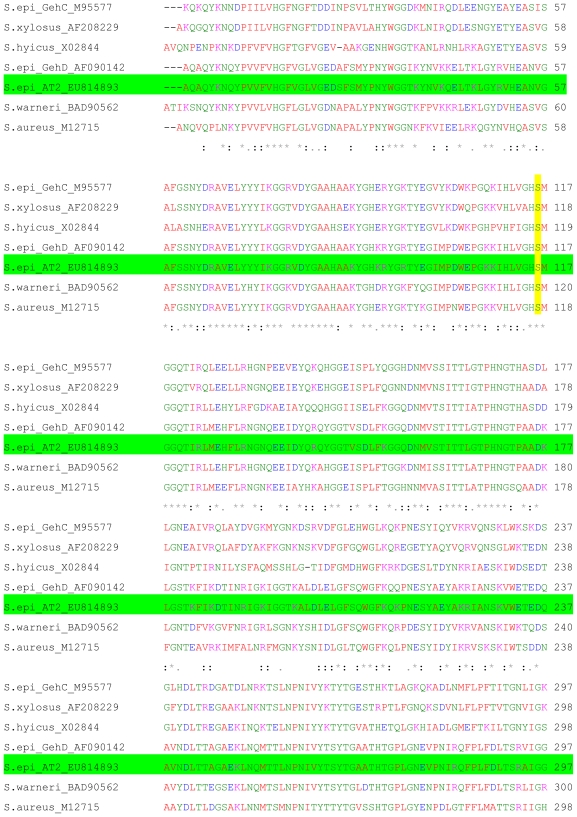

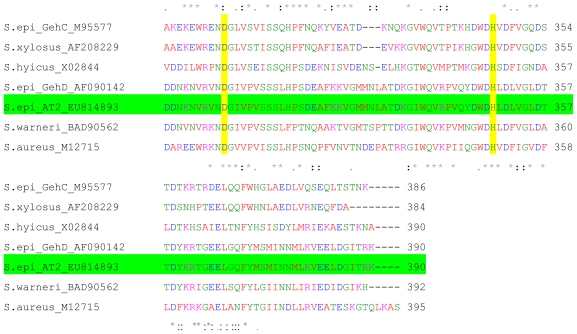
Amino acid sequence alignment of the mature domain of *S. epidermidis* AT2 lipase with those from other Staphylococcal lipases. Note: Identical amino acid residues are indicated in bold type with asterisks below. The conserved serine, aspartic acid and histidine residues, which are predicted to be important in the lipase active site, are highlighted in yellow. Amino acid numbers are shown on the right. AT2 lipase (EU814893) is highlighted in green.

Microbial enzymes held some common elements in the secondary and tertiary structure although they are structurally diverse [9]. The mature lipase region in particular, showed a high degree of conservation between all staphylococcal lipase sequences as shown in Figure 2. Ser-116 in the mature AT2 lipase was located in the conserved G-X-S-X-G sequence around the active site serine residue based on its sequence alignment with those other staphylococcal lipases. This Ser-His-Asp residue was the common catalytic site triad for lipases and also similar was found in serine proteases. The nucleophilic serine was present in the highly conserved pentapeptide motif Gly-X_1_-Ser-X_2_-Gly [10].

**Figure 3:**
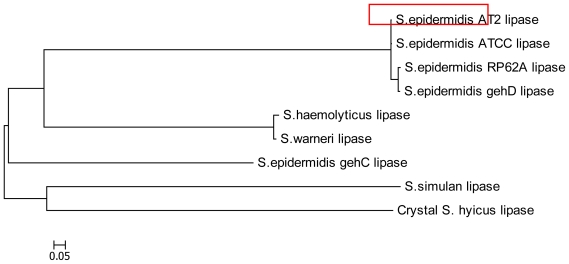
The phylogenetic analysis showed that AT2 lipase is closely related to *S. epidermidis* ATCC lipase, *S. epidermidis* RP62A lipase and lipase *gehD* from *S. epidermidis* 9. Note: The evolutionary distances are in the units of the number of amino acid substitutions per site.

The phylogenetic tree for AT2 lipase was constructed through multiple sequence alignments with other members of *Staphylococcal* lipases by Molecular Evolutionary Genetics Analysis (MEGA4). The result of phylogenetic tree AT2 lipase is represented in Figure 3. AT2 lipase was closely related to lipase ATCC, *gehD* and RP62A. Both *gehD* and *gehC* were originated from *S. epidermidis* 9, however, within the *Staphylococcal* lipase family, the two lipases of *S. epidermidis* 9 were grouped into separately branched clusters.

### 2.2. Expression of AT2 Lipase in Prokaryotic System

The mature lipase gene was successfully cloned and expressed in pTrcHis2, an expression vector carrying IPTG inducible *trc* promoter, with *E. coli* TOP10 as the host. Optimization of the IPTG concentration was carried out and resulted in an increase of lipase activity by 500% upon the induction of 0.1 mM IPTG compared to the non-inducible culture (data not shown).

Enhancement of the activity was observed when a higher concentration of IPTG was applied to the recombinant clones, and reached its maximum activity of 5.3 U/mL, a 16-fold increase at 0.6 mM IPTG. At higher concentrations of IPTG (>0.6 mM), the lipase activity gradually dropped. Over production and accumulation of the gene products probably caused misfolding of the enzymes, and thus promoted the formation of inclusion bodies and aggregations [13]. In the expression of soluble cytoplasmic recombinant proteins, the optimal IPTG concentration for *trc* or other derived promoters were widely reported to be around 1.00 mM. However, for production purposes, a small amount of IPTG was sufficient to ensure the economical feasibility [14].

The post induction time-course study was also investigated at 0.6 mM IPTG induction (data not shown). At 0 h induction time, lipase activity detected was very low (0.36 U/mL). A significant increase was observed after 4 h of induction (2.11 U/mL) and increased continuously up to 10 h of induction with 5.58 U/mL. The expression level decreased gradually after 20 to 24 h with 32% loss in lipase activity. The outgrowing of non-induced cells containing plasmid in the later phase could have caused the gradual drop of the expression level.

This result suggested that the AT2 mature lipase gene was successfully cloned and expressed in *E. coli* system with high level of protein expression (~18-fold) compared to its wild type. Lipase from *S. haemolyticus* L62 which had been successfully expressed in pBluescript II SK (+) in *E. coli* XL1 Blue; showed a low expression level although the *N*-terminal signal sequence of the preproenzyme was correctly removed [15]. In another case, the expression of *S. hyicus* lipase in *Lactococcus lactis* had lead to a 10-fold increase of lipolytic activity [16].

### 2.3. SDS PAGE Analysis

**Figure 4:**
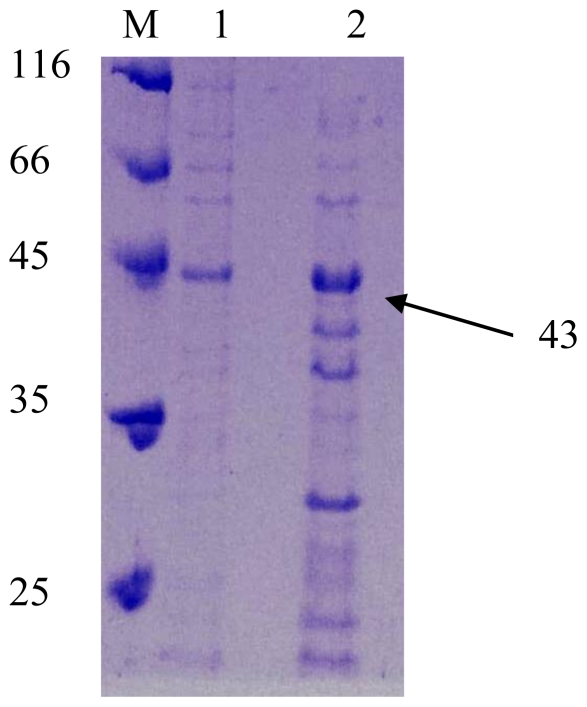
SDS PAGE analysis of AT2 in (1) the absence of 0.6 mM IPTG as inducer; (2) and its presence; (M) Protein molecular weight marker. The expression product was electrophoresed on 12% (w/v) of SDS-PAGE and stained with Coomassie Blue.

Based on the sequence comparison, literature showed that all staphylococcal lipases were primarily synthesized as pre-pro lipase. Many staphylococcal lipases were reported to produce a ~80-kDa preproenzyme and secreted into the culture medium as a ~45-kDa mature form due to proteolytic processing [15]. In this study, the mature AT2 lipase was detected by SDS-PAGE analysis. The molecular weight of this mature lipase as predicted by ExPASy (Expert Protein Analysis System) (http://expasy.org/tools/) was 43.6 kDa. In this regard, Figure 4 revealed a ~43 kDa protein of the recombinant AT2 lipase that was successfully expressed.

### 2.4. Stability of Recombinant Lipase in Various Organic Solvents

AT2 lipase was identified as an organic solvent stable enzyme in the previous study. The stability of recombinant AT2 lipase in the presence and absence of organic solvents was determined. The enzyme was treated for 30 min in 25% (v/v) organic solvents and assayed for lipase activity. The relative activity after 30 min incubation in 25% (v/v) of organic solvent is as shown in Table 1. Log P was used as the quantitative measure of the solvent polarity. It is the logarithm of the partition coefficient, P, of the solvent in a defined 1-octanol-water mixture [17]. In the presence of water miscible organic solvent AT2 lipase did not retain its activity except for dimethylsulfoxide (DMSO), with 112% of the relative activity. In agreement, purified recombinant lipase from *B. sphaericus* 205y was also reported to be activated in the presence of DMSO by ~50% increase in the activity [18] while lipase from *P. aeroginosa* LST-03 showed high stability in 25% (v/v) DMSO for 15 days [19]. Purified lipase from *Cryptococcus* sp. S-2, a yeast, was also activated by 5–10% (v/v) DMSO [20]. In contrast, the crude lipase of *B. sphaericus* 205y was inhibited by DMSO, as well as purified recombinant lipase from *P. fluorescence* JCM6963 [21,22]. Water miscible organic solvents had a greater tendency to inactivate lipases and esterases. The enzymatic activity was affected due to the direct contact of the organic solvent with the enzyme [23].

Organic solvents with log *P* between 1.5 to 4, such as benzene (log *P* = 2.0), toluene (log *P* = 2.5), octanol (log *P* = 2.9) and n- hexane (log *P* = 3.5), were very toxic to microorganisms as they could accumulate in the cell membrane thus, causing cell disruption [24]. Interestingly, AT2 lipase was greatly activated by some water immiscible solvents including toluene, octanol, p-xylene and n-hexane to more than ~50% increment. Lipase from *S. saprophyticus* M36 showed a similar finding except for its stability in the presence of benzene [25]. By contrast, AT2 lipase showed a reduction of activity by ~40% in benzene. The interaction of hydrophobic solvent and hydrophobic amino acid residues, present in the lid of the enzyme, enhanced the activity as the lid was in its open conformation [23,26].

The stability of enzymes was suggested to be influenced by the solvent polarity; however, correlations between a simple parameter such as log *P* and an even more complicated factor such as denaturation capacity, can never exactly predict the effects of solvents on enzymes in general. There are large individual variations among enzymes and no particular trend of the inactivating effect of the organic solvents towards enzymes [27–29].

## 3. Experimental Section

### 3.1. Sources of Bacteria

A pure culture of *S. epidermidis* AT2 was obtained from stock culture from Enzyme and Microbial Technology laboratories, UPM. It was previously isolated from contaminated soil at a car service station in Port Dickson, Negeri Sembilan.

### 3.2. DNA Manipulation

Genomic DNA from *S. epidermidis* AT2 was prepared according to standard procedures with some modification [30]. Plasmid DNA was isolated with a QIAGEN miniprep spin kit (QIAGEN; Hilden, Germany) according to the manufacturer’s instructions. The PCR product was purified with GeneClean Kit (Qbiogene; Carlbad, USA) as described by the manufacturer. Competent cells of *E. coli* TOP 10 was prepared by using a conventional calcium chloride method.

### 3.3. Sequencing and Analysis of Lipase Gene

The recombinant plasmid was sequenced with an ABI PRISM 377 DNA automated sequencer (Applied Biosystem, USA). The nucleotide sequence of AT2 lipase gene was identified and deposited into Genbank under accession number EU814893. The comparison of the DNA sequence was carried out by using the database of the National Centre of Biotechnology (http://www.ncbi.nih.gov). Meanwhile, the analysis of the lipase gene was achieved with Biology Workbench (http://www.biology.ncsa.sdsc.edu) and ExPASy (Expert Protein Analysis System) (http://www.expasy.org.tools)

### 3.4. Cloning of an Organic Solvent-Tolerant AT2 Lipase Gene

A set of primers were designed based on the sequence of *S. epidermidis gehD* (AF090142) conserved region.

FOR: 5′-CAG TGG TCA GCA TGC TCA AGC-3′

REV: 5′-GCT AGG TTC ATC ATA CCT ACC TTC-3′

The part of the gene encoding the mature lipase was amplified by using PCR from genomic DNA of *S. epidermidis* AT2. A set of degenerated primer was designed based on sequence from *S. epidermidis* lipase precursor (*gehD*)(AF090142).

Fm: 5′-CAA TCA ACT TAC TGC GCA AGC-3′

Rm: 5′-CAC TAC TTA CGT GTG ATA CCA-3′

Amplification process was carried out in a reaction mixture containing 50–100 ng DNA template, 30 pmol each forward and reverse primers, 0.2 mM dNTP mix, 2 mM MgCl_2_, 2U *Taq* DNA polymerase (MBI Fermentas; St. Leon-Rot, Germany), 10× PCR buffer with the following PCR conditions: an initial denaturation step at 94 °C for 4 min, 35 cycles at 94 °C for 1 min, annealing at 45 °C for 1 min, and extension at 72 °C for 1 min, except for the final extension of 7 min, and preservation at 4 °C.

The PCR product was electrophoresed using 1% (w/v) agarose gel and purified by using a Gel Extraction kit (Qiagen, USA). The purified PCR product was cloned into pGEM-Teasy vector (Promega, USA) according to the manufacturer’s instructions and transformed into *E. coli* Top 10 competent cells. The positive clones were obtained on the screening agar; tributyrin-LB agar plate (100 μg/mL ampicillin), and further confirmed on triolein and rhodamine B-LB agar (100 μg/mL ampicillin) plates. The positive transformants were then screened by using colony PCR.

### 3.5. Expression of AT2 Lipase with pTrcHis2 TOPO TA

For expression purposes, the purified PCR product of the mature lipase gene region was cloned into pTrcHis2 TOPO TA expression vector (Invitrogen; Groningen, Netherlands), and transformed in *E. coli* Top 10 competent cells. The positive colony was cultured in LB broth, at 37 °C, overnight. The culture harboring recombinant plasmids; pTrcHis2/AT2 was then inoculated in a 1 L blue cap bottle containing 200 mL sterilized LB broth supplemented with 100 μg/mL ampicillin and incubated on a rotary shaker (200 rpm) at 37 °C. IPTG (0.6 mM) was added at OD_600nm_ ~ 0.5 for 10 h. The culture (10 mL) was harvested by centrifugation and resuspended with 2 mL of 50 mM phosphate buffer (pH 7) before sonication (Branson 250 sonifier: output 2, duty cycle 30 and 2 min) and cleared by centrifugation (8500 × *g*, 20 min). The clear crude lysate was assayed for lipase activity according to the Kwon and Rhee method [31].

### 3.6. Lipase Activity Assay

Liberated free fatty acids were determined by calorimetric method using olive oil as a substrate. An equal volume of olive oil (Bertoli, Italy) and 50 mM phosphate buffer (pH 7) was mixed to prepare the emulsion. One milliliter of enzyme was added to 2.5 mL of the emulsion plus 20 μL of 0.02 M CaCl_2_ and was shaken at 200 rpm for 30 min at 37 °C. One milliliter of 6 N HCl and 5 mL of isooctane were then added to stop the enzyme reaction. This was followed by a vigorous mixing for 30 s with a vortex mixer. Four milliliters of the upper isooctane layer was transferred to a test tube containing 1 mL of copper reagent. The copper reagent was prepared by 5% (w/v) copper (II) acetate-1-hydrate and the pH was adjusted to 6.1 with pyridine. The absorbance of the upper layer which contained the liberated fatty acids was read at 715 nm. Lipase activity was determined by measuring the amount of free fatty acid released by referring to the standard curves of free fatty acids. One unit of lipase activity was defined as the rate of 1 μmol of fatty acid released per minute.

### 3.7. Electrophoresis

SDS PAGE was performed on 12% running gels based on the Laemmli’s method [32]. A broad range of protein standard markers (MBI Fermantas; St. Leon-Rot, Germany) were used as the molecular weight markers.

### 3.8. Effect of Organic Solvents on Crude Enzyme Stability

One milliliter of organic solvent was added to 3 mL of the crude cell lysate and preincubated at 37 °C, with an agitation at 150 rpm for 30 min to ensure the continuous mixing of the enzyme and solvent. The enzyme stability was expressed as the remaining activity assayed according to the Kwon and Rhee (1986) method [31] relative to the control value. Distilled water was used instead of solvent as the control.

## 4. Conclusions

An organic solvent tolerant lipase from *S. epidermidis* AT2 was successfully cloned and expressed in a prokaryotic system with higher activity compared to its wild type. Generally, thermostable enzymes showed a positive correlation with the stability in organic solvents [33]. Interestingly, this recombinant AT2 lipase, a mesophilic enzyme, exhibited a good tolerance and stability towards organic solvents, whereby the activity was significantly activated by 25% (v/v) water immiscible organic solvents mainly of octanol, toluene, *p*-xylene and n-hexane after 30 minutes incubation at 37 °C. This organic solvent tolerant lipase will be useful in a variety of biotechnological fields such as catalysis in organic synthesis, biotransformation and optical resolution of chiral compounds.

## Figures and Tables

**Table 1:** Effect of various organic solvents on AT2 lipase stability.

Solvent	Log P*	Relative activity (%)
Control	-	100
Dimethylsulfoxide	−1.3	112
Methanol	−0.76	1.2
Acetonitrile	−0.33	0
Ethanol	−0.24	0
Acetone	−0.23	0
Diethylether	0.68	0
Ethylacetate	0.68	15
Chloroform	2.0	14
Benzene	2.0	56
Toluene	2.5	230
Octanol	2.9	295
Ethylbenzene	3.1	58
p-xylene	3.1	190
n-hexane	3.5	154

* Adapted from Laane *et al.*, 1987 [17]

Note: Three mL of the crude enzyme was incubated with 1 mL of organic solvent (3:1 ratio) at 37 °C with shaking at 150 rpm for 30 min and the remaining lipase activity was assayed.
